# Homeopathy for Depression - DEP-HOM: study protocol for a randomized, partially double-blind, placebo controlled, four armed study

**DOI:** 10.1186/1745-6215-12-43

**Published:** 2011-02-14

**Authors:** Ubiratan C Adler, Stephanie Krüger, Michael Teut, Rainer Lüdtke, Iris Bartsch, Lena Schützler, Friedericke Melcher, Stefan N Willich, Klaus Linde, Claudia M Witt

**Affiliations:** 1 Institute for Social Medicine, Epidemiology and Health Economics; Charité University Medical Center; D-10098 Berlin, Germany; 2 Clinic for Psychiatry and Psychotherapy, Charité University Medical Center; D-10098, Berlin, Germany; 3 Karl and Veronica Carstens-Foundation; Am Deimelsberg 36; D-45276 Essen; Germany; 4 Institute of General Practice, Technische Universität München, Wolfgangstr. 8, D-81667, Munich, Germany

## Abstract

**Background:**

Homeopathy is often sought by patients with depression. In classical homeopathy, the treatment consists of two main elements: the case history and the prescription of an individually selected homeopathic remedy. Previous data suggest that individualized homeopathic Q-potencies were not inferior to the antidepressant fluoxetine in a sample of patients with moderate to severe depression. However, the question remains whether individualized homeopathic Q-potencies and/or the type of the homeopathic case history have a specific therapeutical effect in acute depression as this has not yet been investigated. The study aims to assess the two components of individualized homeopathic treatment for acute depression, i.e., to investigate the specific effect of individualized Q-potencies versus placebo and to investigate the effect of different approaches to the homeopathic case history.

**Methods/Design:**

A randomized, partially double-blind, placebo-controlled, four-armed trial using a 2 × 2 factorial design with a six-week study duration per patient will be performed. 228 patients diagnosed with major depression (moderate episode) by a psychiatrist will be included. The primary endpoint is the total score on the 17-item Hamilton Depression Rating Scale after six weeks. Secondary end points are: Hamilton Depression Rating Scale total score after two and four weeks; response and remission rates, Beck Depression inventory total score, quality of life and safety at two, four and six weeks. Statistical analyses will be by intention-to-treat. The main endpoint will be analysed by a two-factorial analysis of covariance. Within this model generalized estimation equations will be used to estimate differences between verum and placebo, and between both types of case history.

**Discussion:**

For the first time this study evaluates both the specific effect of homeopathic medicines and of a homeopathic case taking in patients with depression. It is an attempt to deal with the challenges of homeopathic research and the results might be useful information in the current discussion about the evidence on homeopathy

**Trial registration:**

ClinicalTrials.gov: NCT01178255

## Background

From 120 million people that suffer from depression around the world, less than 25% receive adequate treatment [1]. The estimated global burden of disease from major depression, measured as "disability adjusted life years" (DALY), is rising globally, making depression the leading cause of DALYs in middle and high income countries [2]. The overall prevalence of depressive disorders in five European countries was 8.56%, with a higher prevalence in urban Ireland (12.3%) and urban United Kingdom (UK, 17.1%) [3]. The prevalence can be higher in some age groups. For instance, in Berlin, Germany, the prevalence of lifetime depression in 2008 was almost 20% for women between 18 and 29 years old, 25% for women between 40 and 59 years old and, among men, 12% and 15% for the same age groups, respectively [4]. In the same year, depression was the main reason for work incapacity and for early retirement among women [4]. This might be in spite of current treatments, which do not seem to have any effect on reducing the number of disabled persons per year [5].

According to the S3-Guidelines from the German Society for Psychiatry, Psychotherapy and Neurology, an antidepressant treatment is indicated to patients presenting a moderate episode of major depression [6]. Although antidepressants are the standard pharmacotherapy for major depression, with a significant difference to placebo, the National Institute of Clinical Excellence (NICE) in the UK stresses that the severity of depression at which antidepressants show consistent benefits over placebo is poorly defined, emphasizing that, in general, the more severe the symptoms, the greater the benefit [7]. In moderate depression, for instance, there is evidence suggesting that there is a statistically important difference favouring Serotonin Selective Reuptake Inhibitors (SSRIs) over placebo on reducing depression symptoms as measured by the Hamilton Depression Rating Scale (HAM-D), but the size of this difference is unlikely to be of clinical importance (SMD = -0.28; 95% CI, -0.48 to -0.08) [7] A recent patient-level meta-analysis confirms these guidelines and previous data [8], indicating small antidepressant benefits for patients with mild (SMD -0.11; 95% CI -0.18 to 0.41) or moderate depressive episodes (SMD -0.17; 95% CI-0.08 to 0.43 [9]). Whereas, for patients with severe depression, the difference was with a SMD of 0.47 (95% CI, 0.22 to 0.71), which is very close to 0.50, i.e., a medium effect size [9].

The patient's discontentment with antidepressants is a reason cited for the search for other treatment options [10]. In Ireland, for instance, individuals with a history of depression were much more likely to seek complementary and alternative medicine (CAM) than those who were not depressed [11]. Depression is also one of the most commonly treated complaints at the outpatient clinics of homeopathic hospitals in the UK National Health Service [12].

Homeopathy is based on the 'principle of similars', whereby substances that cause symptoms in healthy individuals are used to stimulate healing in patients who have similar symptoms when ill [13]. These substances are usually administered in extremely high dilutions, making homoeopathy a controversial and strongly debated system. When a single homoeopathic remedy is selected based on a patient's total symptom picture, it is called 'classical' homoeopathy [14].

In classical homeopathy (addressed in this protocol) the treatment consists of two main elements: the case history and the prescription of an individually selected homeopathic remedy. The homeopathic case history aims to ascertain the totality of signs and symptoms of each patient, enabling the selection of an individualized homeopathic medicine. In addition it attempts to understand the patient's background, environment and daily routine. In some recent approaches the patient might fill in a questionnaire prior to the medical consultation, to improve the efficiency of obtaining the case history [15].

Homeopathic medicines are produced through sequential agitated dilutions in Decimal (D), Centesimal (C) or Quinquagintamillesimal (Q or LM) potencies. In this study we will use Q-Potencies which are prepared by grinding the raw material (C1 until C3), followed by consecutive 1:50.000 agitated dilutions. Therefore a Q1 corresponds to a 5 × 10^-10 ^fraction of the raw material (Q2 = 2.5 × 10^-15^, Q3 = 1.25 × 10^-20^, Q4 = 6.25 × 10^-24^, etc.). To date, there is no clear evidence that homopethic medicines are superior to placebo.

Results from a multi-center observational study with 3981 patients treated with classical homeopathy indicate clinically relevant improvements in the mental aspects of quality of life, after two and eight years of treatment observation [16]. However, it is not clear whether these results can be attributed to the homeopathic treatment itself, or are due to other factors or just placebo effects since the study had no control group. However, according to a recent trial from Brazil [17], classical homeopathy (i.e., the "whole packet": a thorough case history + homeopathic medicines) seems to be, at least, as effective as conventional standard pharmacotherapy. Data from this randomized, controlled, double-blind trial indicated that individualized homeopathic Q-potencies were non-inferior to the antidepressant fluoxetine in a sample of patients with moderate to severe depression. Interestingly responder rates (defined as a decrease of at least 50% from baseline on the Montgomery & Åsberg depression rating scale) of both the homeopathic medicine and fluoxetine groups were higher (homeopathy 84.6%; fluoxetine 82.8%) than those usually found for antidepressants in trials (43-75%) [18]. One might speculate that this higher response rate could be due to the more extensive homeopathic case history in the study that compared homeopathic medicines with fluoxetine. However, neither the specific effect of individualized homeopathic Q-potencies, nor the specific effect of the homeopathic consultation has been investigated for patients with depression.

### Aims

The primary objective of this study is to assess the two main components contributing to the individualized homeopathic acute phase (6 weeks) treatment of depression (moderate episode), i.e., to investigate the specific effect of individualized Q-potencies versus placebo and to investigate the effect of different forms of taking a homeopathic case history (case history type I and II).

Secondary objectives will be to investigate short term effects (after two and four weeks), including differences between individualized Q-potencies and placebo as well as between the different techniques of homeopathic case history, and to assess the effect of individualized Q-potencies together with either form of case history. A safety evaluation will also be performed.

## Methods/Design

### Study Design

A randomized, partially double-blind, placebo-controlled, four-armed trial using a 2 × 2 factorial design with a six week study duration per patient will be performed to test two different hypotheses:

H_0_: homeopathic medicines = placebo (null hypothesis) vs. H_1_: homeopathic medicines ≠ placebo (alternative hypothesis)

H_0_: homeopathic case history type I = case history type II (null hypothesis) vs.H_1_: homeopathic case history type I ≠ case history type II (alternative hypothesis)

**Figure 1:**
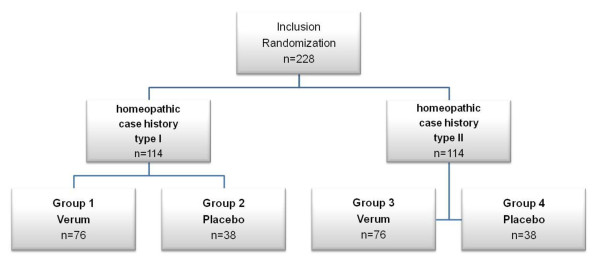
**Flow chart of the study groups**.

To test these hypotheses, patients will be randomized to one of four groups illustrated in Figure 1.

### Participants

We will include male and female patients aged between 18 and 65 years diagnosed with moderately severe (HAM-D 17 to 24) major depression by a psychiatrist, patients must not be currently taking antidepressants or anxiolytic drugs (with the exception of Lorazepam as rescue medication, maximal dose 1.5 mg/day). Capability and willingness to give informed consent and to comply with the study procedures will also be required.

Exclusion criteria include current mild episode of depression (HAM-D < 17), current severe episode of depression (HAM-D > 24); schizophrenia or other psychotic disorders, bipolar affective disorder, schizoaffective disorders, alcohol or other substance abuse, eating disorders, a clinically significant (Diagnostic and Statistical Manual of Mental Disorders)-Axis II disorder; severe depression, which previously motivated a suicide attempt; a score of 4 or 5 in the Columbia-Suicide Severity Rating Scale (C-SSRS) [19], up to three months before screening; a clinically significant acute or chronic disease that would hinder regular participation in the study; treatment with antipsychotics, antidepressants, sedatives/hypnotics or mood stabilizers four weeks prior to the screening; complementary or alternative treatment simultaneously to the study (for example, acupuncture, phytotherapy, etc.); homeopathic treatment eight weeks prior to study entry; psychotherapy; simultaneous participation in another clinical trial (the last participation in a previous clinical trial must be completed at least three months prior to screening); concomitant pregnancy or breastfeeding; patients who are assumed to have a linguistic, intellectual or any other reason for not understanding the meaning of the clinical trial and for not complying with the necessary study procedures; persons who have been institutionalized by a court order; patients with an application for a pension.

Participants will be interviewed and treated by a medical doctor specialized in homeopathy at the CHAMP outpatient clinic of Charité University Medical Center.

### Interventions

#### Homeopathic case history - Type I or II

After inclusion, patients will be randomly assigned to either case history type I or II, according to a randomization number disclosed from sequentially numbered, sealed opaque envelopes. This involves disclosing information using different homeopathic techniques, in order to assess their influence on depression severity. Type I and II differ in the time used for the semi-standardized questionnaire and the onsite patients-doctor interaction and to ensure patients' blinding are not further described here. The content and structure of the questionnaire used in DEP-HOM follows Hahnemann's case history instructions [20], with some additional questions stressing the symptoms of a depressive episode. The questionnaire was developed in consensus with members of the German Homeopathic Doctors Association and pretested.

#### Individualized homeopathic Q-potencies or placebo

The selection of the individualized remedy (case analysis) will be carried out after the case history, in the absence of the patient, by medical doctor specialized in homeopathy with 20 years experience classical homeopathy based on the the clinical-pharmaceutical protocol [21] developed by Hahnemann, which includes the standardized use of ascending Q-potencies [20]. The investigator is also experienced in case history and analysis under double blind conditions [17].

Q-potencies will be provided from the study pharmacy by Dr. Zinsser Arzneimittel, (Freudenstadt, Germany) and are manufactured according to the methodology described in the 6^th ^edition of the *Organon. *The prescription of the individualized homeopathic Q-potency will be sent to the Charité pharmacy, together with the patient's randomization number. According to the randomization number, the study pharmacist will dissolve one sucrose globule of the prescribed Q-potency (Q2) or one sucrose globule (placebo) in 10 ml of 20% alcohol-distilled water solvent. The vial will then be labeled and sent to the study center, responsible for dispatching it to the patient within three days from the first case history.

The standard dose will be one drop of the received vial three times per week [21]. Follow-ups will be at two, four and six weeks after the first clinical interview. Blinded medicine, dosage or potency changes will be allowed on a clinical basis. Basal Q-potency medicines stored by the study Pharmacy are listed below (Appendix 1).

### Outcomes

The primary endpoint is the mean total depression score using the 17-item version of the Hamilton Depression Rating Scale (HAM-D) [22], after six weeks. Severity of symptoms will be assessed by a blinded investigator (psychologist) from the Clinic for Psychiatry and Psychotherapy, Charité University Medical Center. The secondary end points are the mean HAM-D total scores after two and four weeks, response (decrease of 50% or more from baseline HAM-D score) and remission (HAM-D scores ≤ 7) rates, Beck Depression inventory (BDI) [23] total score and mean SF-12 Health Survey (SF-12) at weeks two, four and six.

Adverse events will be collected during the study and will form part of the secondary endpoint data in determining the safety of homeopathic medicines. Serious adverse effects from homeopathic medicine were not observed on the non-inferiority trial [17] and are not expected during the current study. Participants' treatment expectations at baseline will also be assessed.

### Randomization and blinding

A non-stratified block randomization with variable block lengths will be carried out, with a 2:1:1:2 ratio (aiming at exposing a smaller number of participants to placebo treatment) for group 1: group 2: group 3: group 4 (i.e. 76:38:38:76 patients). The randomization list was generated with SAS/BASE Software (SAS Inc., Cary NC, USA), by a statistician not further involved in the study. The patients will be assigned in sequential order to the treatment groups.

The patients, the psychiatrist and the statistician will remain blinded from the identity of the four treatment groups until the end of the study. The study clinical investigator will be unmasked for the case history type I or II. The randomization list will be kept strictly confidential. Only the study pharmacist and the statistician who generated the randomization list have access to the randomization list. During the study, unblinding will only occur in the case of a patient emergency using sealed emergency envelopes.

### Data management

Data management services will be performed by the study center at the Institute for Social Medicine, Epidemiology and Health Economics - Charité University in accordance with the ICH-Guidelines for Good Clinical Practice and DIN EN ISO 2001.

### Statistical Analysis

The following primary comparisons will be conducted between the following groups: The specific effect of homeopathic medicines compared to placebo (double blind comparison), where essentially the groups 1+3 will be compared to the groups 2+4. The effect of the homeopathic case history (single-blind comparison), where essentially groups 1+2 (case history type I) are compared to the groups 3+4 (case history type II).

#### Analysis for the primary endpoint

Statistical analysis will be by intention-to-treat, including all patients randomized, regardless whether or not they adhered to the treatment protocol or provided complete data sets. Only patients who withdraw their consent to use their personal data can be excluded from the analysis.

To indicate, whether or not the randomisation process leads to prognostically balanced treatment groups, all baseline parameters will be compared by two sided Chi-square-tests (if nominally scaled) or Kruskal-Wallis-tests (if ordinally or continuously scaled). The respective p-values are descriptive in nature, not confirmative.

The primary endpoint will be analysed by a two-factorial analysis of covariance (ANCOVA), modeling time (3 levels: weeks 2, 4 and 6) as a within-group-factor, type of case history (2 levels: types I and II), type of medicine (2 levels: verum and placebo), and their respective interaction as between-group factors, and baseline value and patient's expectation as linear covariates. Within this model generalized estimation equations (GEE) [24] will be used to estimate the 6-week differences between verum and placebo, and between both types of case history. Two-sided p-values and confidence intervals for both hypotheses will be adjusted by the Bonferoni-Holm procedure [25]. As no interim analyses are planned there is no need for further multiple adjustments. The multiple level of significance is set at α = 0.05 (two-sided).

#### Sample size calculation

For this study we assumed that the verum treatment is better than placebo by 2.7 ± 6.0 (mean ± standard deviation) HAM-D score points after 6 weeks (corresponding to a SMD = 0.45), that type II case history is better than type I by 2.7 ± 6.0 score points (SMD = 0.45), and that both effects do not interact. If so, a Bonferoni-adjusted F-Test (multiple significance level α = 0.05, two-sided) has a power of 83.5% to detect the difference between verum and placebo and a power of 85.0% to detect the difference in case history taking, if 68 patients are included in groups 1 and 3, and 34 patients are included in groups 2 and 4. This leads to a total number of 228 patients, if one allows for a 10%drop-out rate per group.

### Regulatory and Ethical approval

#### Regulatory approval

Bundesinstitut für Arzneimittel und Medizinprodukte (BfArM), EudraCT Nr: 2009-017458-11, Submission-Nr.: 4036175.

#### Ethical approval

Ethics Committee, Berlin, Landesamt für Gesundheit und Soziales (LaGeSo): ZS EK 15 099/10. This study is in compliance in with the Helsinki Declaration and with the International Conference on Harmonisation (ICH) - Good Clinical Practice.

## Discussion

For the first time this study evaluates both the specific effect of homeopathic medicines and of a homeopathic case taking in patients with depression. The protocol is in accordance with the EMEA (European Medicines Agency) Guidelines, which recommends placebo-controlled studies and the duration of six weeks for trials investigating medicines for depression [26], considering that during antidepressant pharmacotherapy, one must reckon with a delay of several weeks until sufficient antidepressant effects can be seen [27].

It is the first trial on classical homeopathy after the 15^th ^amendment to the German Medicines Act (Arzneimittelgesetz, AMG) [28]. The study includes all relevant aspects of the CONSORT guidelines for reporting randomized homeopathic trials with parallel groups [29,30]. For ethical reasons, individuals with a previous suicide attempt or a C-SSRS score of 4 or 5 will be excluded and the occurrence of suicide ideation (with the same C-SSRS severity) will determine the premature termination of the patient's participation in the study. Depression severity will be limited to a maximum HAM-D score of 24, because for more severe depression a treatment with antidepressant is recommended [9].

The need of individual prescriptions in classical homeopathy has been considered as 'a severe obstacle for any double-blind trial' [31]. In fact, the selection of a suitable, individualized homeopathic medicine will not be always accomplished during the six weeks of acute treatment, especially under double-blind conditions. However, from an ethical point of view a longer placebo treatment period is problematic.

This study is an experimental study with a focus on efficacy and not a pragmatic trial with a focus on effectiveness. It is an attempt to deal with the challenges of homeopathic research [32] and the results might be useful information in the current discussion about the evidence on homeopathy.

## Abbreviations

AMG: Arzneimittelgesetz; ANCOVA: analysis of covariance; BDI: Beck Depression Inventar; BfArM: Bundesinstitut für Arzneimittel und Medizinprodukte; CAM: complementary and alternative medicine; C-SSRS: Columbia Suicide Severity Rating Scale; DALY: disability adjusted life years; EMEA: European Medicines Agency; GEE: Generalized Estimated Equation; HAM-D: Hamilton-Depressions-Skala; ICH: International Conference on Harmonisation; LAGeSo: Landesamt für Gesundheit und Soziales; NICE: National Institute of Clinical Excellence; Q-Potenzen: QuinquagintamillesimalPotencies; SMD: Standardized Mean Difference; SF 12: Short Form 12; SSRIs: Serotonin Selective Reuptake Inhibitors.

## Competing interests

The authors declare that they have no competing interests.

## Authors' contributions

CMW, KL, SNW, MT and UCA participated in the design of the study. CMW, SK, KL and UCA reviewed and discussed current data on antidepressants for moderate depression and the ethical basis for a placebo controlled study on homeopathy for depression. CMW, IB, FM, LS and UCA elaborated all study documents, including those necessary for regulatory and ethical approval. RL performed the statistical planning. CMW, KL, MT, RL, SK and UCA helped to draft the manuscript. All authors read and approved the final manuscript.

## Appendix 1

Q-potencies that will be stored at the Charité Pharmacy (Q2 and Q3). Medicines not listed can optionally be ordered and prescribed, as needed.

*Agaricus muscarius, Alumina, Ammonium carbonicum, Ammonium muriaticum, Anacardium orientale, Antimonium crudum, Arsenicum album, Aurum foliatum, Baryta carbonica, Borax, Calcarea aceticum, Calcarea carbonica, Carbo animalis, Carbo vegetabilis, Causticum, Cocculus indica, Colocynthis, Conium maculatum, Digitalis, Graphites, Hepar sulphuris calcareum, Ignatia amara, Iodium, Kalium carbonicum, Kalium nitricum, Lycopodium clavatum, Magnesia carbonica, Magnesia muriatica, Manganum, Mercurius solubilis, Mezereum, Muriaticum acidum, Natrum carbonicum, Natrum muriaticum, Nitri acidum, Nux vomica, Petroleum, Phosphoricum acidum, Phosphorus, Platina, Pulsatilla pratensis, Rhus toxicodendron, Sepia succus, Silicea terra, Spigelia, Stanum, Staphisagria, Sulphur, Sulphuricum acidum, Zincum*.
